# An alien in Marseille: investigations on a single *Aedes aegypti* mosquito likely introduced by a merchant ship from tropical Africa to Europe[Fn FN1]

**DOI:** 10.1051/parasite/2022043

**Published:** 2022-09-16

**Authors:** Charles Jeannin, Yvon Perrin, Sylvie Cornelie, Andrea Gloria-Soria, Jean-Daniel Gauchet, Vincent Robert

**Affiliations:** 1 Entente Interdépartementale pour la Démoustication du littoral Méditerranéen 165 avenue Paul Rimbaud 34184 Montpellier Cedex 4 France; 2 MIVEGEC unit, Univ. Montpellier, IRD, CNRS 34394 Montpellier Cedex 5 France; 3 IRD-Délégation régionale Occitanie 911 avenue Agropolis BP 64501 34394 Montpellier Cedex 5 France; 4 Department of Entomology, Center for Vector Biology & Zoonotic Diseases, The Connecticut Agricultural Experiment Station 123 Huntington St. New Haven CT 06511 United States; 5 Department of Ecology and Evolutionary Biology, Yale University 21 Sachem Street New Haven CT 06520-8105 United States

**Keywords:** Mosquito, Propagule, Invasive species, Point of entry, Surveillance

## Abstract

Control of invasive species relies partly on permanent surveillance at international points of entry. We report the exceptional trapping of one adult mosquito (Diptera: Culicidae) in the port of Marseille, France, in July 2018, during a routine survey conducted according to International Health Regulations. Morphological and molecular identification classified the specimen as a female *Aedes* (*Stegomyia*) *aegypti* (L.), vector of many arboviruses, absent from Europe and the Mediterranean rim since the 1950s. A world reference panel of approximately 23,000 genome-wide single nucleotide polymorphisms determined that the mosquito originated from Cameroon, west Africa. Cross-reference of this geographic location with boats traveling from Central Africa to Marseille during the trapping period suggests that the mosquito travelled within an identified merchant ship, a vehicles carrier connecting Douala, Cameroon to Marseille, France. This ship left Douala on June 25, 2018 and arrived 20 days later in Marseille on July 15. The mosquito was captured 350 m away from the dock. The interception of a propagule of an invasive species is a rare event that must be considered a priority to prevent its successful establishment.

## Abstract (fr)

La lutte contre les espèces invasives repose en partie sur une surveillance permanente aux points d’entrée internationaux. Nous rapportons ici le piégeage exceptionnel d’un moustique adulte (Diptera: Culicidae) dans le port de Marseille, France, en juillet 2018, au cours d’une enquête de routine menée selon les recommandations du Règlement Sanitaire International. L’identification morphologique et moléculaire a désigné ce spécimen comme étant une femelle d’*Aedes* (*Stegomyia*) *aegypti* (L.), vecteur de nombreux arbovirus, absent d’Europe et du pourtour Méditerranéen depuis les années 1950. Une base de référence mondiale du polymorphisme des nucléotides individuels pour ~23 000 génomes complets a permis de déterminer que ce moustique était originaire du Cameroun. Le croisement de cette information de localisation géographique avec celle de la circulation des bateaux entre l’Afrique Centrale et Marseille au cours de la période de piégeage suggère que le moustique a voyagé à l’intérieur d’un navire de commerce identifié, un transporteur de véhicules reliant Douala (Cameroun) à Marseille (France). Ce navire a quitté Douala le 25 juin 2018 pour arriver à Marseille 20 jours plus tard, le 15 juillet 2018. Le moustique a été capturé à 350 mètres du dock. L’interception d’un propagule d’une espèce invasive est un évènement rare qui doit être considéré de façon prioritaire afin d’empêcher la réussite de son installation.

## Introduction

Specific measures to prevent the introduction of vectors and vector-borne diseases are implemented in France to satisfy the International Health Regulation (IHR). As described in its revised version of 2005, the purpose of IHR [45] is “to prevent, protect against, control and provide a public health response to the international spread of disease”. Regarding vector-borne diseases, the aim is to stop the dissemination of vectors by implementing surveillance and control measures at international points of entry (ports, airports, and ground crossings). These measures were transcribed into the National law of France in 2013 (https://solidarites-sante.gouv.fr/fichiers/bo/2014/14-09/ste_20140009_0000_0036.pdf). They concern mosquitoes and are mandatory in regions where the vector *Aedes albopictus* (Skuse) is established, which is the case along the entire Mediterranean coast of France. These measures are implemented by the platform administrator under the control of the French regional health agency of the district.

This study reports the detection of a female *Aedes* (*Stegomyia*) *aegypti* (L.) at the Port of Marseille in July 2018. Interestingly, this report echoes an account by Aubert and Guérin [3] following the detection of a specimen of *Stegomyia fasciata* (the name of the species at that time) at the Parc du Pharo in Marseille on November 22, 1907. Here, modern research tools allowed us to pinpoint the origin of the mosquito and the likely importing boat.

Because *Ae. aegypti* is the main vector of yellow fever and dengue viruses [14], and also a vector of the chikungunya [29] and Zika viruses [23], the establishment of this mosquito on the Mediterranean coast would have a significant health impact.

## Materials and methods

### Study site, surveillance and vector control program

The “Grand Port Maritime de Marseille” (GPMM) on the Mediterranean coast of France includes two sites, the western and eastern docks. With 81 million tons of goods in 2018, it is the main port in France and ranks second in the Mediterranean Sea. It also ranks as the number one cruise port in France and is among the top five in the Mediterranean Sea, with three million passengers in 2018 (https://www.marseille-port.fr/sites/default/files/2020-12/Rapport_Annuel_2019.pdf). The study area concerns only the eastern docks of the GPMM, extending over an area of 400 ha along 8 km of coast in Marseille city (43°20′31″ N, 5°20′10″ E). Activities in the eastern dock include passenger transport with ferries and cruises, goods transport with liquid and solid-bulk transport, and container transport with roll-on/roll-off container ships (Ro-Ro)*,* lift-on/lift-off container ships (Lo-Lo), ships with both (Con-Ro) and roll-on/roll-off-passenger ships (Ro-Pax) (Online material 1.1).

The invasive mosquito species *Ae. albopictus* was introduced into France from Italy in 2004 and was detected in Marseille city in 2009. By 2018, this arboviral vector was widely established throughout the city (https://solidarite-sante.gouv.fr). An entomological evaluation conducted in the port and the surrounding environment within a radius of 400 metres in 2015 by a local public mosquito control operator (EID Méditerranée, operator of the platform administrator) revealed high abundance of *Ae. albopictus* and guided the surveillance and control program for the next year. This program includes larval surveillance, control of the potential breeding sites, and surveillance of adult mosquitoes with mosquito trap networks.

### Mosquito sampling and morphological identification

Since 2016, the *Ae. albopictus* population is monitored by a network of 4 Mosquito Magnet Independence traps (MM-trap) (Woodstream Corp, Lititz, PA, USA; http://www.mosquitomagnet.com) for host-seeking females and a network of 10 Gravid Aedes Traps (GAT) (Biogents, GmbH, Regensburg, Germany, https://biogents.com) for gravid females.

The MM-trap attracts host-seeking females by producing CO_2_ and hot water vapour. Both Lurex (L-Lactic acid) and Octenol (1-octenol-3-ol) cartridge baits (Woodstream Corp, Lititz, PA, USA; http://www.mosquitomagnet.com) are added to optimize the attractiveness of the trap [19, 20, 38]). Many studies have demonstrated the ability of these traps to capture a wide variety of mosquito species in a variety of environments, and particularly, a high number of container-inhabiting *Aede*s species such as *Ae. albopictus*, *Ae. aegypti* and *Ae. japonicus* (Theobald) [19, 28, 30].

The GAT trap is a passive trap that utilizes olfactory cues from water and visual cues to attract and trap gravid mosquitoes [18]. The trap is composed of a black bucket (with water), a translucent plastic chamber and a black funnel on the top. A black nylon mesh excludes mosquito from the water source. It is constructed so females enter a funnel into the chamber and fly toward the light in an attempt to escape this dark and confined space (“fly to the light” concept) [11, 37]. It has also been demonstrated to be effective at catching the vectors *Ae. aegypti* and *Ae. albopictus* and is complementary to the CO_2_ trap [7, 11, 18, 37].

Both trap networks were continuously operated (24/7) between May 4 and November 2, 2018, outdoors, in shaded, wind-protected moist areas, as far as possible from each other (Online material 1.2). Every four weeks, trapped mosquitoes were collected, traps were maintained, and baits refilled. Mosquitoes were morphologically identified using the keys of Schaffner *et al.* [39] and MosKeyTool [16].

Following the French guidelines of the surveillance and control program (https://solidarites-sante.gouv.fr/IMG/pdf/SurveillanceControle_des_vecteurs_V2_BD.pdf), the introduction of invasive mosquitoes requires implementation of complementary measures as follows: (i) addition of adult traps monitored every two weeks, (ii) inspection of potential resting sites with mouth-aspirator, (iii) search for larvae in potential breeding sites, and (iv) mosquito control (larvicide and adulticide if necessary). The two larvicides used for the control of the non-removable breeding sites are the Vectobac^®^ G (Sumitomo Chemical; active substance: *Bacillus thuringiensis* var. *israelensis*, Strain AM65-52, serotype H14) and a silicone-based surface film, Aquatain™ Mosquito Formulation (AMF, Dobol^®^).

### Molecular identification (PCR and/or sequencing)

DNA extraction from the abdomen was performed with CTAB, as described previously [46]. DNA was resuspended in 20 μL of water. DNA concentration was evaluated with the Nanoquant at 79 ng/μL. Approx. 400 bp and 900 bp fragments of the NADPH 4 Subunit (ND4) and cytochrome oxidase 1 (COI) genes, respectively, were amplified individually using primers described elsewhere [22]).

For ND4, PCR amplification was performed in 25 μL total volume containing 4 μL of 1/20 template DNA dilution, 2.5 μL of 10× reaction buffer, 1.5 mM MgCl_2_, 0.2 mM dNTP, 20 pmol of each primer and 1 UI of Taq polymerase (Eurogentec). PCR was conducted at 95 °C for 3 min, followed by 35 cycles at 94 °C for 30 s, 50 °C for 30 s and 72 °C for 30 s with a final extension at 72 °C for 5 min.

For the COI gene, the reaction mix contained 6 μL of 1/20 template DNA dilution, 2.5 μL of 10× reaction buffer, 1.5 mM MgCl_2_, 0.2 mM dNTP, 20 pmol of each primer and 1 UI of Taq polymerase (Eurogentec). PCR was conducted at 95 °C for 3 min, followed by 35 cycles at 94 °C for 1 min, 54 °C for 30 s and 72 °C for 1 min with a final extension at 72 °C for 10 min.

PCR products were identified by agarose gel electrophoresis stained with Midori^green^ advance (Nippon genetics). All positive PCR products were sent to Eurofins Genomics for Sanger sequencing in both directions. Sequences were aligned with Muscle (http://www.drive5.com/muscle) as implemented in MEGA X (https://www.megasoftware.net/). COI sequences were also directly submitted to Boldsystems (www.barcodeoflife.org) for blast analysis.

### Genotyping and population genetic analysis

DNA was extracted from the thorax and head of the intercepted *Ae. aegypti* individual using a Qiagen DNeasy Blood and Tissue Kit (Qiagen, Hilden, Germany), following the manufacturer’s instructions, with an additional RNAse A step. Genotyping was performed using an Axiom_aegypti1 SNP chip (Life Technologies Corporation, Carlsbad, CA, USA; CAT#550481 [12]) at the University of North Carolina Functional Genomics Core, Chapel Hill, NC, USA. This genotyping array was designed from representative populations of *Ae. aegypti* around the world to best capture the genetic variation of this species and allows the identification of *Ae. aegypti* subspecies, *aegypti* or *formosus* [12, 24]. Additional data from previously described samples from populations of *Ae. aegypti* collected worldwide were used as a reference panel (Online material 1.3). Loci that fail to genotype at 80% or more of the individuals from the global dataset were filtered out from the 22,849 loci obtained from the SNP-chip that met Mendelian expectations, using the – geno 0.2 option in plink 1.9 ([6]; www.cog-genomics.org/plink/1.9/). Subsequently, individuals with more than 5% missing data were removed with the – mind 0.05 option. The final dataset had 22,845 SNPs and 784 individuals from across *Ae. aegypti* distribution (including the individual from Marseille). Genetic assignment tests of the *Ae. aegypti* from Marseille against the worldwide reference dataset were performed in GeneClass2 [34]. Previous studies have shown higher accuracy of this assignment method using SNPs [13, 24]. Ten independent runs were conducted with sets of 3500 SNPs drawn at random using the command – thin-count 3500 from PLINK 1.9. ([6]; www.cog-genomics.org/plink/1.9/), and the Bayesian criteria for likelihood estimation to determine the population-assignment ranking [36]. Self-assignment tests on the SNP reference dataset resulted in 97.98 ± 0.22% of individuals assigned to the expected countries.

### Tracking of candidate vessel carriers

**Figure 1:**
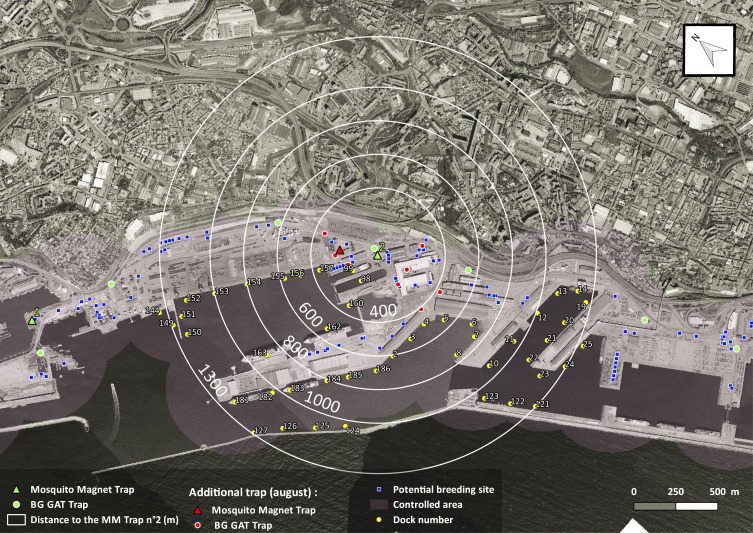
Map of the eastern dock of the “Grand Port Maritime de Marseille” (GPMM), France with location of traps, breeding sites and docks.

Information on boats arriving in the port of Marseille from May to July was requested from the port authorities, including widely the period of capture of the *Ae. aegypti* specimen. *Aedes aegypti* is not a good flyer [15], so we restricted our search to vessels that had docked up to a distance of 1300 m from the place of capture. Consequently, every dock used during the trapping period was plotted on a map and distances from the place of capture calculated using QGIS 2.18 (Quantum Geographic Information System) software (Fig. 1). Due to the unknown variability of the berthing locations, the orientations of the boats along the dock and their large size, we used distance classes to which we assigned coefficients inversely proportional to the distance to the place of capture. The coefficient and the trapping index formula are presented in Online material 2.

The most helpful information, i.e., countries of origin of the vessels, was absent from the database; only the last stopover was mentioned. To fill this gap, a database of the regular shipping lines was built and analyzed to identify the riskiest lines. Thus, for each shipping line, an import risk index was established according to different parameters: travel time, number of transhipments and presence of *Ae. aegypti* in the country of origin. The last parameter received different coefficients depending on the presence of the species in the country, according to the following references ([10, 17, 25], ECDC mosquito-maps 2019; https://www.ecdc.europa.eu/en/disease-vectors/surveillance-and-disease-data/mosquito-maps). The risk index is zero if the vector is not present in the country. The travel time and number of transhipments was obtained from the websites of shipping companies. In the calculation of the risk index, travel time and the number of transhipments decrease the risk. The coefficients and the import index formula are presented in Online material 3.

## Results

The trapping network captured 5,608 indigenous mosquitoes between May 4 and November 2, 2018: 72% *Cx. pipiens* L., 21% *Ae. albopictus* (Skuse), and 7% miscellaneous (Table 1). In addition, one adult female mosquito suspected of belonging to *Ae. aegypti* was captured in July (between June 29 and July 23) in MM-trap number 2, located close to a building situated near the docks “Med Europe”, “Léon Gourret” and “Cap Janet” (43°20′35.74″ N, 5°20′39.12″ E). No more *Ae. aegypti* adult specimens were captured in the nine additional traps (eight GAT traps and one MM-Trap) set up after the detection (August 3) and until October, or during the resting site inspection (August 8–9). Larval sampling carried out on August 8–9 over an area of 40 ha around the detection site detected two breeding sites containing Culicidae larvae. The sites were both treated with AMF (a catch basin, and a telecommunication manhole chamber). These two larval sites contained exclusively indigenous mosquitoes including *Cx. pipiens* [pupae (*n* = 4), larvae (*n* = 45)] and *Ae. albopictus* [pupae (*n* = 2), larvae (*n* = 35)].

**Table 1:** Number of mosquitoes caught during the surveillance period by species and month.

	May	June	July	August	September	October	Total	Proportion (%)
*Aedes aegypti*	0	0	1	0	0	0	1	0.02
*Aedes albopictus*	31	111	353	424	233	51	1204	21.47
*Aedes caspius*	6	13	4	3	0	4	30	0.53
*Aedes detritus*	7	3	0	0	0	8	18	0.32
*Aedes vexans*	0	0	0	3	0	0	3	0.05
*Culex pipiens*	1101	1418	731	561	175	68	4054	72.28
*Culex modestus*	0	215	0	4	0	0	219	3.90
*Anopheles maculipennis*	1	0	0	0	0	0	1	0.02
*Culiseta longiareolata*	7	23	38	0	10	0	78	1.39
*Uranotaenia unguiculata*	0	0	1	0	0	0	1	0.02
Total	1153	1783	1128	995	418	131	5609	100.00

Morphological observation identified the individual as *Ae. aegypti*, but the specimen was slightly damaged, without the typical scale pattern on the scutum (Online material 1.4). In a first attempt to confirm the morphological identification, two sets of *Ae. aegypti* primers were used to amplify the ND4 and COI genes. A band of the right size was recorded for both PCRs, strongly suggesting that the specimen belonged to *Ae. aegypti* species. Sequencing and subsequent Blast query confirmed the specimen as *Ae. aegypti* with an e-value < 1e^−171^ for the ND4 fragment and an e-value of 0.0 for the COI fragment. A query on Boldsystems returned more than 20 hits of *Ae. aegypti* with 100% similarity, confirming the species identification of the specimen caught in Marseille.

Individual genetic assignment tests (GeneClass2) using ten different subsets of 3500 SNPs derived from the SNP chip, identified the subspecies as *Ae. aegypti formosus* and Cameroon as the likely source of the introduction (8/10). The rest of the tests assigned the individual to a population from Burkina Faso (2/10).

The arriving vessel database reported 406 dockings and included 138 different boats. Thirty-two local boats (docked 190 times) were excluded. The 216 remaining dockings (107 boats) were assigned a range of trapping indices between 0.1 and 5 (Online material 2). Among them, the 20 riskiest vessels that docked closest to the place of capture during the trapping period belonged to 5 companies and are shown in Table 2.

**Table 2:** Twenty riskiest boats docked between May and July 2018.

Boat name	Kind of boat	Company name	Dock number	Last stopover	Arrival date
Grande togo	Con-Ro	Grimaldi acl fr	157	Valencia (ES)	30/06/2018
Mississauga expr	Container	Clients divers	F08	Sagunto (ES)	03/07/2018
Pacaya	Container	Cma cgm sa	157	Ghazaouet (AL)	06/07/2018
Repubblica del brasile	Con-Ro	Grimaldi acl fr	157	Sète (FR)	15/07/2018
Jolly quarzo	Ro-Ro	Ignazio messina	157	Barcelona (ES)	16/07/2018
Grande costa d’avorio	Con-Ro	Grimaldi acl fr	157	Valencia (ES)	21/07/2018
Leto	Container	Cma cgm sa	155	Algeciras (ES)	01/07/2018
Susan borchard	Container	Borchard lines	156	Salerno (IT)	03/07/2018
Katherine borchard	Container	Borchard lines	155	Barcelona (ES)	04/07/2018
Hsl nike	Container	Cma cgm sa	156	Genova (IT)	05/07/2018
Okee ann mari	Container	Cma cgm sa	156	Genova (IT)	06/07/2018
Joanna borchard	Container	Borchard lines	156	Barcelona (ES)	07/07/2018
Benedikt rambow	Container	Cma cgm sa	156	Oran (AL)	07/07/2018
Joanna borchard	Container	Borchard lines	156	Barcelona (ES)	10/07/2018
Miriam borchard	Container	Borchard lines	156	Barcelona (ES)	11/07/2018
Erato	Container	Cma cgm sa	155	Genova (IT)	12/07/2018
Jaguar	Container	Cma cgm sa	156	Valencia (ES)	12/07/2018
Jolly quarzo	Ro-Ro	Ignazio messina	155	Barcelona (ES)	15/07/2018
Saumaty	Cargo	Marfret	156	Alger (AL)	18/07/2018
Winchester strait	Container	Cma cgm sa	155	Genova (IT)	20/07/2018

The regular shipping lines database compiled 196 lines with a range of import risk indices between 0 and 122 (Online material 3). The 10 riskiest shipping lines concerning the same 5 companies and 7 areas are presented in Table 3. The maritime lines linking West Africa and the GPMM are the most represented and one of them links Senegal and the GPMM in a minimum of 6 days. After comparison of the 10 riskiest shipping lines and the 20 riskiest vessels, we identified one boat as the prime suspect responsible for the import of the intercepted *Ae. aegypti* specimen. This boat left Douala, Cameroon on the June 25 and arrived in Marseille on July 15 (20 days later). It docked at dock 157 located 350 m away from the point of capture (Mosquito Magnet No. 2), eight days before trap collection. The 19 remaining candidate boats do not link West Africa to the GPMM or have transhipment in the city of Algeciras, Spain.

**Table 3:** Twenty riskiest shipping lines in connection with the port of Marseille. Con-Ro and Lo-Lo are defined in the materials and methods.

Zone	Company name	Type of boat	Origin country	Minimum travel time (days)
West Africa	GRIMALDI	Con-Ro	Senegal	6
East Africa	MESSINA	Con-Ro	Djibouti	10
West Africa	GRIMALDI	Con-Ro	Nigeria	12
Middle East	MESSINA	Con-Ro	Saudi Arabia	12
Caribbean	CMA CGM	Lo-Lo	Guadeloupe	13
West Africa	GRIMALDI	Con-Ro	Benin	14
West Africa	GRIMALDI	Con-Ro	Ivory Coast	15
West Africa	GRIMALDI	Con-Ro	Togo	15
West Africa	GRIMALDI	Con-Ro	Ghana	16
West Africa	GRIMALDI	Con-Ro	Cameroon	16
South America	CMA CGM	Lo-Lo	Colombia	18
Central America	CMA CGM	Lo-Lo	Costa Rica	19
Middle East	CMA CGM	Lo-Lo	Egypt	20
Central America	MARFRET	Lo-Lo	Costa Rica	20
South America	MARFRET	Lo-Lo	Colombia	20

## Discussion

New invasive mosquitoes are making their way into Europe at an accelerated pace, all introduced by humans via the transport of goods and travellers. Three invasive mosquitoes of the genus *Aedes* are currently established in Europe: *Ae. japonicus*, *Ae. koreicus* (Edwards), and *Ae. albopictus*. *Aedes atropalpus* (Coquillett) and *Ae. triseriatus* (Say) have also been introduced into the Netherlands and France but have not yet become established [31]. *Aedes aegypti* is not established in Europe but is established in Turkey (Asian part), Georgia and Madeira (see below) and documented worldwide in Asia, the Americas and Africa [35].

This is the first detection of *Aedes aegypti* in Marseille since 1908 [3], an introduction that was not followed by establishment [4]. *Aedes aegypti* was established and abundant along the French Riviera in the Alpes-Maritimes including the city of Nice around 1917 [4]. The species, although not established, was also detected along the French Mediterranean coast in Hyères [43] around 1902 and Bastia, Corsica around 1925 [27]. The detections in a boat in Brest [26] and in Bordeaux [43] at the beginning of the 20th century and epidemics of dengue (Cadiz, 1784; Athens, 1928) and yellow fever (Cadiz, 1800; Barcelona, 1821; Lisbon, 1857) also attest to multiple importations into European ports throughout the years [32, 40].

Disappearance of *Ae. aegypti* from the region between the 1960s and 2000 seems to coincide with the establishment of piped water supply systems that reduced the availability of larval habitats and the intensive use of DDT (dichlorodiphenyltrichloroethane) for malaria control programs [40]. Since the 1950s, the species was incidentally detected in Italy [5], Israel [33] and sporadically in Turkey, suggesting the persistence of small populations over time [8, 41]. Today, populations have re-established in Madeira [44], around the Black Sea [2, 24], and in Egypt [1]. In Europe, the species was detected in the Netherlands in 2010 at a used tire importer [42], in England far from any port or airport, near Liverpool in 2014 [9], and in the Netherlands in 2016 at Schiphol Airport [21] based on results contained through the IHR.

The design and analysis of the regular routes linking the GPMM allowed the identification of the most probable routes of introduction of *Ae. aegypti* into this port. The most represented routes originated in West Africa. The mosquito intercepted was identified as an adult *Ae. aegypti* native of the area around Cameroon, likely transported from Douala. The travel times of ships from this area are compatible with the life expectancy of an adult mosquito (20 days in this case). Unfortunately, we do not have information confirming the presence of larval sites or appropriate developmental conditions for *Ae. aegypti* within the boat, because inspection of the boat did not take place due to the time that it took to conclude the investigation. However, since very few containers open directly on the port docks (survey conducted with the port authorities of some transport companies), it is likely that the captured female did not come from within a container but rather from the hold of the boat. Short travel times would have optimized the exit of individuals directly to the port of Marseille. The probability that the captured female is the result of eggs laid in the port of Marseille following a previous introduction is low. No specimens have been caught during the intense surveillance that followed the interception or during the monthly inspection of potential breeding sites performed for larval suppression, strongly suggesting that the trapped *Ae. aegypti* is a propagule.

Identification of the source of the *Ae. aegypti* introduction to Marseille was possible through the molecular analysis of this mosquito and the availability of a global genetic reference panel of *Ae. aegypti*, narrowing the origin down to the geographic region of Cameroon. Importantly, the possibility of a point of origin in Burkina Faso may be ruled out due to the continental isolation of the country. The Cameroonian origin guided the subsequent investigation by the mosquito control operator and pointed to the potential boat responsible for the introduction. These results could now be used to inform future surveillance by choosing the capture sites closest to the docks used by the riskiest lines and by adapting the mosquito collection schedule to the dates of arrival of the riskiest ships in order to optimize detection of exotic vectors.

Inspections of the riskiest boats could also be carried out to detect the different developmental stages (adults, larvae, eggs) that may be transported and to evaluate the survival capacity of this vector during travel in the boat.

The temperature thresholds for the persistence of *Ae. aegypti* populations are thought to be the January isotherm of 10 °C or the annual mean temperature of 15 °C [40]. In our case, January isotherms in Marseille are around 8 °C, and in January 2019 mean temperature was 7.3 °C. These values are above the extremes of temperature range of *Ae. aegypti*, which makes its establishment almost impossible in theory. However, the species is established in the United States in 26 states [17], some of which have negative temperatures in winter like Washington, DC where an overwintering population has been described [28].

Mosquito control operators must be aware that a single *Ae. aegypti* among hundreds, if not thousands, of *Ae. albopictus* may remain undetected by microscopic observation. A careful visual inspection keeping *Ae. aegypti* in mind is required. It could be asked whether complementary genetic analysis of pools of collected mosquitoes may provide interesting results.

## Conclusion

The detection of one *Ae. aegypti* mosquito in Marseille highlights a possible route of introduction of the *Ae. aegypti* vector into Europe and illustrates the importance of a quick response to implement appropriate vector control measures. It also demonstrates the need to set up a surveillance system for invasive mosquitoes at points of entry, including ports, and how genetic analyses and a complete genetic reference panel for the species can support control efforts.

## Supplementary materials

The Supplementary materials of this article are available at https://www.parasite-journal.org/10.1051/parasite/2022043/olm. *Online material 1*: 1.1 Map of the eastern dock of the GPMM and mode of shipping according to the docks; 1.2. Map of the eastern dock of the GPMM with location of traps and breeding sites; 1.3. Populations included in the global genetic panel of *Ae. aegypti* used in the present study; and 1.4. Photomicrograph of the specimen *Aedes* (*Stegomyia*) *aegypti* (L.) female collected by the Mosquito Magnet Trap No. 2 in the GPMM between June 29 and July 23, 2018. (PDF file) *Online material 2*: Maritime traffic of the eastern dock of the Grand Port Maritime of Marseille (GPMM) from May 1 to July 31, 2018. Contains the database (boat list with the docking date and the last port frequented), the risk weighting method applied to each vessel and the results of this weighting. (Excel file with 3 sheets) *Online material 3*: Regular maritime lines of the eastern dock of the “Grand Port Maritime de Marseille” (GPMM) in 2018. Contains the database (maritime lines list with the linked ports, the presence of *Aedes aegypti* in the country linked, the travel time and the occurrence of a transhipment), the risk weighting method applied to each maritime line and the results of this weighting. (Excel file with 4 sheets)
