# Supplementary material for: An alien in Marseille: investigations on a single Aedes aegypti mosquito likely introduced by a merchant ship from tropical Africa to Europe
Source: Parasite. 2022 Sep 16;29:42. doi: 10.1051/parasite/2022043 (PMC9479680; doi:10.1051/parasite/2022043)

On line material 1.1 Map of eastern dock of the Grand Port Maritime de Marseille GPMM and mode of shipping according to the docks.

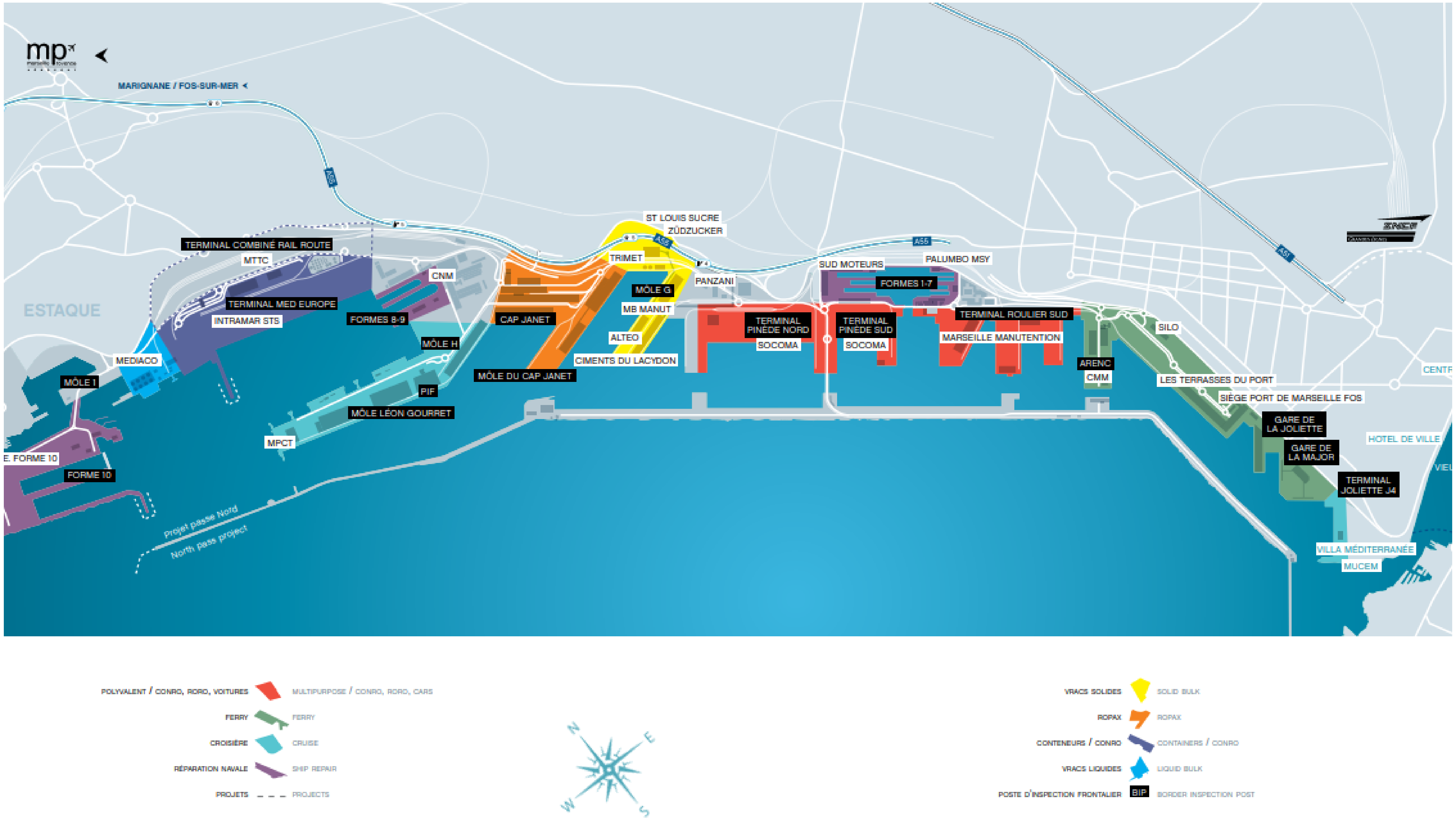

On line material 1.2. Map of eastern dock of the GPMM with location of traps and breeding sites.

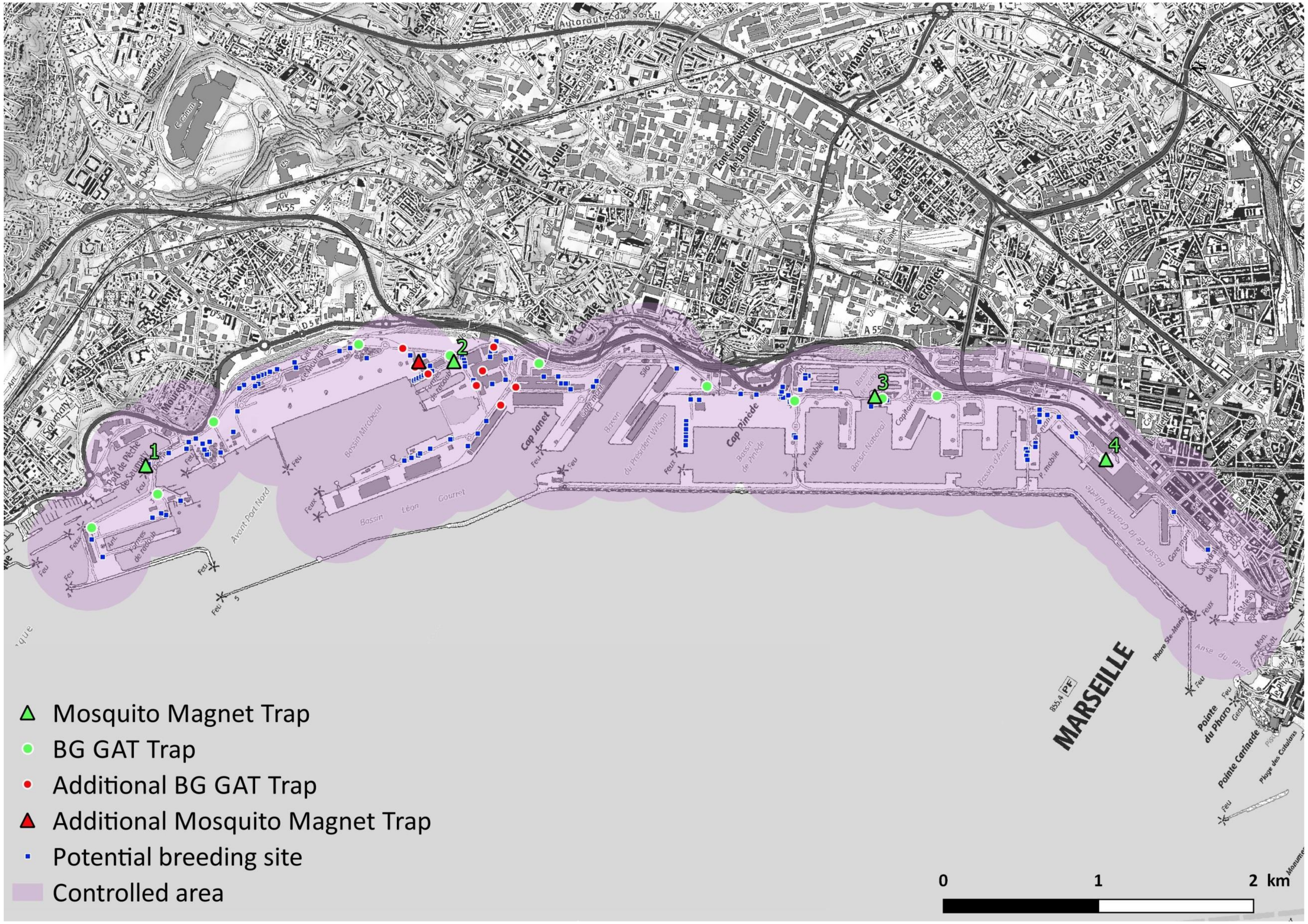

On line material 1.3. Sources of the global database of the the global genetic panel of *Ae. aegypti* used in the present study.

Collection site	Country	Continent	Latitude	Longitud	Year of collection	Reference
Yaounde, Entebbe	Cameroon	Africa	3,866667	11,516667	2014	Kotsakiozi et al. 2018a
Francesville	Gabon	Africa	-1,633243	13,583004	2014	Kotsakiozi et al. 2018a
Lope Forest	Gabon	Africa	−0.37896	11,5274	2014	Kotsakiozi et al. 2018a
Gabon village	Gabon	Africa	-0,1062554	11,60694219	2016	Kotsakiozi et al. 2018a
Gabon forest	Gabon	Africa	−0.37896	11,5274	2016	Kotsakiozi et al. 2018a
Goudiry	Senegal	Africa	14,183	-12,717	2012	Kotsakiozi et al. 2018a
Sedhiou	Senegal	Africa	12,707018	-15,555173	2012	Kotsakiozi et al. 2018a
Johannesburg	South_Africa	Africa	-25,9904	27,9006	2015	Kotsakiozi et al. 2018a and 2018b
Lunyo	Uganda	Africa	0,072055	32,463409	2012	Kotsakiozi et al. 2018a
Jacobina, Bahia	Brazil	Americas	-11,18356	-40,513876	2013	Evans et al. 2015
Jacobina, Bahia	Brazil	Americas	-11,18356	-40,513876	2016	Evans et al. 2019
Macapa	Brazil	Americas	0,035417	-51,071058	2012	Kotsakiozi et al. 2018a
Maraba	Brazil	Americas	-5,361295	-49,11663	2010	Gloria-Soria et al. 2018
Rio de Janeiro	Brazil	Americas	-22,908333	-43,196389	2014	Gloria-Soria et al. 2018
Cali, Valle del Cauca	Colombia	Americas	3,438936	-76,515572	2013	Gloria-Soria et al. 2018
Siquirres	Costa Rica	Americas	9,938477	-84,0953	2014	Gloria-Soria et al. 2018
Nogales, Sonora	Mexico	Americas	31,294114	-110,951837	2013	Gloria-Soria et al. 2018
Tapachula, Chiapas	Mexico	Americas	14,90246	-92,26953	2012	Evans et al. 2015
Tijuana, BCN	Mexico	Americas	32,509466	-116,962878	2013	Gloria-Soria et al. 2018
Madeira	Portugal	Americas	32,666667	-16,916667	2012	Gloria-Soria et al. 2018
Clovis, California	USA	Americas	36,827016	-119,691764	2013	Gloria-Soria et al. 2018
El Paso, Texas	USA	Americas	31,790278	-106,423333	2015	Gloria-Soria et al. 2018
Exeter, California	USA	Americas	36,29619	-119,141919	2014	Pless et al. 2017
Fresno, California	USA	Americas	36,755904	-119,780854	2015	Gloria-Soria et al. 2018
Garden_Grove_2015	USA	Americas	33,779792	-117,952755	2015	Pless et al. 2017
Houston, Texas	USA	Americas	29,755797	-95,367302	2011	Evans et al. 2015
Key West, Florida	USA	Americas	24,556488	-81,780211	2015	Evans et al. 2015
Las Cruces, New Mexico	USA	Americas	32,314444	-106,778889	2015, 2017	Pless et al. 2022
Los Angeles, California	USA	Americas	34,05432	-118,278555	2014	Pless et al. 2017
Madera, California	USA	Americas	36,963744	-120,06937	2013	Gloria-Soria et al. 2018
Madera, California	USA	Americas	36,963744	-120,06937	2015	Pless et al. 2017
Miami, Florida	USA	Americas	25,775163	-80,208615	2011	Pless et al. 2020
New Orleans, Louisiana	USA	Americas	30,0618	-89,900849	2011	Saarman et al. 2017
New Orleans, Louisiana	USA	Americas	30,0618	-89,900849	2015	Evans et al. 2015
North Key West	USA	Americas	24,557342	-81,776735	2013	Gloria-Soria et al. in press
Orlando, Florida	USA	Americas	28,54	-81,38	2014	Pless et al. 2020
San Mateo, California	USA	Americas	37,56121	-122,323784	2013	Pless et al. 2017
Tucson	USA	Americas	32,210246	-110,918824	2012	Gloria-Soria et al. 2018
Washington , DC	USA	Americas	38,889722	-77,011111	2014	Gloria-Soria et al. 2018
Cairns	Australia	Asia_Pacific	-16,925556	145,775278	2013	Kotsakiozi et al. 2018a
Cairns, Queensland	Australia	Asia_Pacific	-16,817295	145,685777	2013	Kotsakiozi et al. 2018a
Tahiti	French_Polynesia	Asia_Pacific	-17,531055	-149,557697	2010	Evans et al. 2015
Attoc & Karachi	Pakistan	Asia_Pacific	33,907222	72,311111	2010	Gloria-Soria et al. 2018
Cebu	Philippines	Asia_Pacific	10,283251	123,946788	2013	Gloria-Soria et al. 2018
Jeddah	Saudi_Arabia	Asia_Pacific	24,71147	46,719532	2012	Gloria-Soria et al. 2018
Sri Lanka	Sri_Lanka	Asia_Pacific	7,423854	80,683227	2014	Gloria-Soria et al. 2018
Bangkok	Thailand	Asia_Pacific	13,7525	100,494167	2011	Gloria-Soria et al. 2018
Milolii, Hawaii	USA	Asia_Pacific	19,2055556	155,8819444	2009	Evans et al. 2015
Hanoi_2013	Vietnam	Asia_Pacific	21,033632	105,842457	2013	Gloria-Soria et al. 2018
Ho Chi Minh City	Vietnam	Asia_Pacific	10,803189	106,694706	2013	Gloria-Soria et al. 2018
Siquirres	Costa Rica	Caribbean	9,748917	-83,753428	2014	Kotsakiozi et al. 2018a
Dominica North Coast	Dominica	Caribbean	15,614399	-61,406879	2016	Gloria-Soria et al. 2018
Roseau	Dominica	Caribbean	15,301389	-61,388333	2016	Gloria-Soria et al. 2018
Trinidad	Republic of Trinidad and Tobago	Caribbean	10,669937	-61,515069	2014	Gloria-Soria et al. 2018
Tobago	Republic_of_Trinidad_and_Tobago	Caribbean	11,25	-60,667	2015	Gloria-Soria et al. 2018
St. Vincent	Saint Vincent and the Grenadines	Caribbean	13,250737	-61,186804	2015	Gloria-Soria et al. 2018
Patillas	USA	Caribbean	18,006792	-66,015716	2014	Gloria-Soria et al. in press
Georgia_2015	Georgia	Europe	41,9614	43,36235	2015	Kotsakiozi et al. 2018b
Hopa, Turkey	Turkey	Europe	39,598918	36,448316	2015	Kotsakiozi et al. 2018b

Rabai	Kenya	Africa	-3,931733	39,571518	2009, 2012, 2017	Kotsakiozi et al. 2018b
Ouagadougou	Burkina Faso	Africa	12,2383	−1.5616	2015	Kotsakiozi et al. 2018a
Buffalo camp	Cameroon	Africa	8,371057	13,866	2014	Kotsakiozi et al. 2018a
Luanda	Angola	Africa	−9.76667	14,26667	2016	Kotsakiozi et al. 2018a
Kaya Forest	Kenya	Africa	−3.93194	39,5961	2012	Kotsakiozi et al. 2018a
Kisimu	Kenya	Africa	−1.19451	36,9456	2012	Kotsakiozi et al. 2018a
Argentina	Argentina	Americas	-34	-64	2006, 2014	Kotsakiozi et al. 2018b
Yaounde Forest	Cameroon	Africa	3,87601	11,3761	2015	Kotsakiozi et al. 2018a
Mokolo	Cameroon	Africa	3,87275	11,5012	2015	Kotsakiozi et al. 2018a
Mvog Ada	Cameroon	Africa	3,86275	11,5259	2015	Kotsakiozi et al. 2018a
Yaounde Village	Cameroon	Africa	3,86076	11,3937	2015	Kotsakiozi et al. 2018a
Zika Village	Uganda	Africa	0,12745	32,5313	2016	Kotsakiozi et al. 2018a

Kotsakiozi et al. 2018a	Kotsakiozi P., Evans B.R., Gloria-Soria A., Kamgang B., Mayanja M., Lutwama J., Le Goff G., Ayala D., Paupy C., Badolo A. & Pinto J., 2018. Population structure of a vector of human diseases: *Aedes aegypti* in its ancestral range, Africa. Ecology and evolution, 8(16), 7835-7848.
Kotsakiozi et al. 2018b	Kotsakiozi P., Gloria-Soria A., Schaffner F., Robert V. & Powell J.R., 2018. *Aedes aegypti* in the Black Sea: recent introduction or ancient remnant? Parasites & Vectors, 11(1), 1-13.
Evans et al. 2015	Evans B.R., Gloria-Soria A., Hou L., McBride C., Bonizzoni M., Zhao H. & Powell J.R., 2015. A multipurpose, high-throughput single-nucleotide polymorphism chip for the dengue and yellow fever mosquito, *Aedes aegypti*. G3: Genes, Genomes, Genetics, 5(5), 711-718.
Evans et al. 2019	Evans B.R., Kotsakiozi P., Costa-da-Silva A.L., Ioshino R.S., Garziera L., Pedrosa M.C., Malavasi A., Virginio J.F., Capurro M.L. & Powell J.R., 2019. Transgenic *Aedes aegypti* mosquitoes transfer genes into a natural population. Scientific Reports, 9(1), 1-6.
Gloria-Soria et al. 2018	Gloria-Soria A., Lima A., Lovin D.D., Cunningham J.M., Severson D.W. & Powell J.R., 2018. Origin of a high-latitude population of Aedes aegypti in Washington, DC. The American journal of tropical medicine and hygiene, 98(2), 445.
Gloria-Soria et al. in press	Gloria-Soria A., Faraji A., Hamik J., White G., Amsberry Sh., Donahue M., Buss B., Pless E., Veiga Cosme L. & Powell J.R. Origin of high latitude introductions of *Aedes aegypti* to Nebraska and Utah during 2019. Infection, Genetics, and Evolution. In press.
Pless et al. 2017	Pless E., Gloria-Soria A., Evans B.R., Kramer V., Bolling B.G., Tabachnick W.J. & Powell J.R., 2017. Multiple introductions of the dengue vector, *Aedes aegypti*, into California. PLoS neglected tropical diseases, 11(8), e0005718.
Pless et al. 2020	Pless E., Powell J.R., Seger K.R., Ellis B. & Gloria-Soria A., 2022. Evidence for serial founder events during the colonization of North America by the yellow fever mosquito, *Aedes aegypti*. Ecology and evolution, 12(5), e8896.
Saarman et al. 2017	Saarman N.P., Gloria-Soria A., Anderson E.C., Evans B.R., Pless E., Cosme L.V., Gonzalez-Acosta C., Kamgang B., Wesson D.M. & Powell J.R., 2017. Effective population sizes of a major vector of human diseases, *Aedes aegypti*. Evolutionary Applications, 10(10), 1031-1039.

On line material 1.4. Photomicrograph of the specimen *Aedes* (*Stegomyia*) *aegypti* (L.) female collected by the Mosquito Magnet Trap N°2 in the GPMM between June 29 and July 23, 2018.

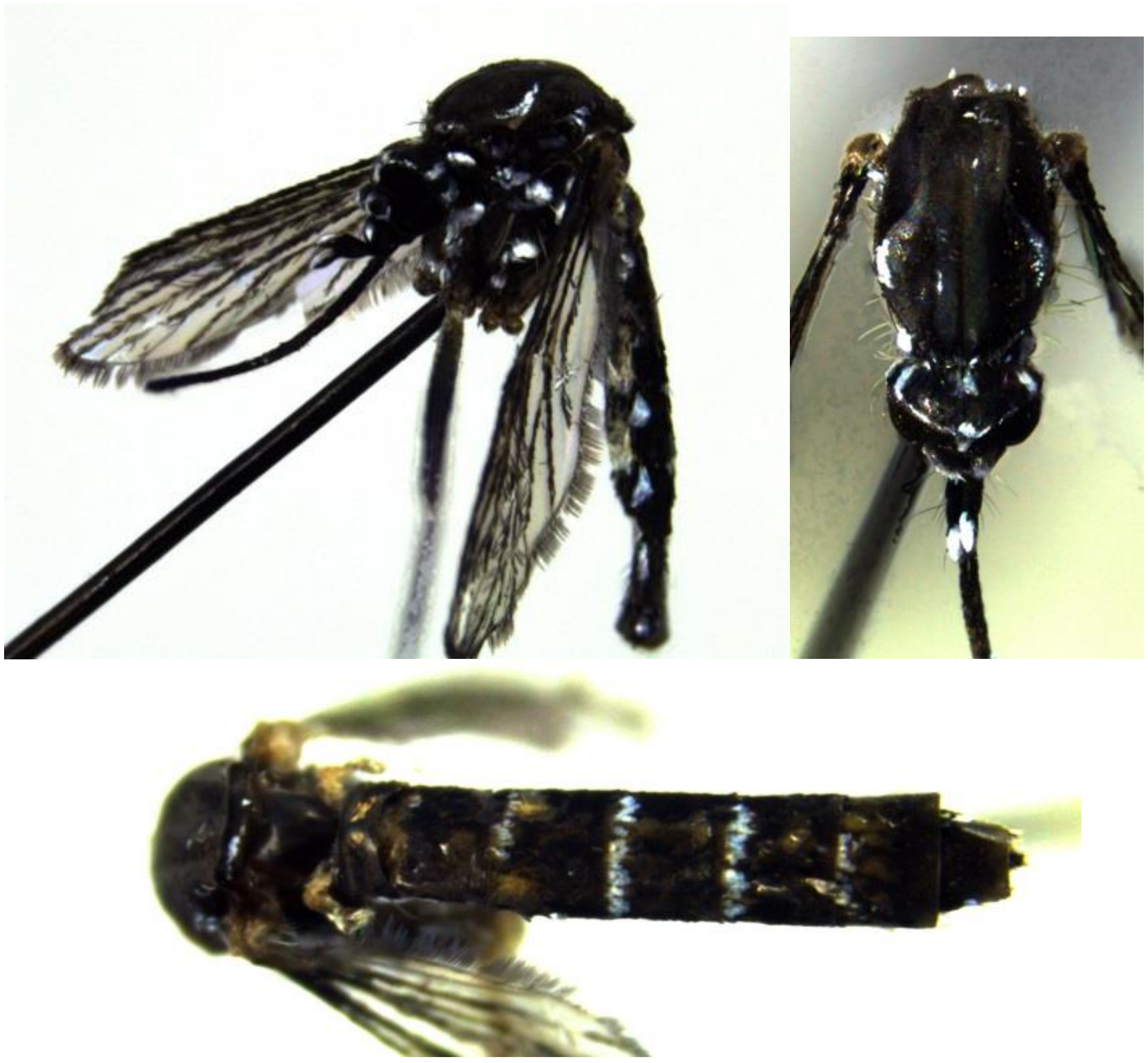

Supplement: Online material 1: — 1.1 Map of the eastern dock of the GPMM and mode of shipping according to the docks; 1.2. Map of the eastern dock of the GPMM with location of traps and breeding sites; 1.3. Populations included in the global genetic panel of Ae. aegypti used in the present study; and 1.4. Photomicrograph of the specimen Aedes (Stegomyia) aegypti (L.) female collected by the Mosquito Magnet Trap No. 2 in the GPMM between June 29 and July 23, 2018. (PDF file) [file parasite-29-42-s1.pdf]
